# Specificity and retention of visual perceptual learning in young children with low vision

**DOI:** 10.1038/s41598-020-65789-1

**Published:** 2020-06-01

**Authors:** Bianca Huurneman, F. Nienke Boonstra, Jeroen Goossens

**Affiliations:** 1 Radboud University Medical Centre, Donders Institute for Brain, Cognition and Behaviour, Cognitive Neuroscience Department, Nijmegen, The Netherlands; 2 Royal Dutch Visio, Nijmegen, The Netherlands

**Keywords:** Perception, Paediatric research, Attention, Consolidation

## Abstract

There is evidence that a pen-and-paper training based on perceptual learning principles improves near visual acuity in young children with visual impairment. The aim of the present study is to measure specificity and retention of its training effects during one year. Sixteen visually impaired children aged 4–8 years were divided in two age- and acuity-matched groups: an early (n = 9) and late treatment group (n = 7). Training consisted of 12 sessions (2× per week for 6 weeks). Studied variables were uncrowded and crowded binocular near visual acuity (40 cm), distance visual acuity (3.0 m) and fine motor skills (Beery VMI, subtest Motor Control). In the early treatment group, we measured at 0 months (pre-training), at 2 months (post-training), at 8 months (6 months post-training) and at 14 months (12 months post-training) since inclusion. In the late treatment group, three pre-training measurements were performed at 0, 2 and 8 months, and two measurements at 0 and 6 months post-training. In the short term, training improved uncrowded and crowded near visual acuity at 0.4 m by 0.13 ± 0.03 and 0.09 ± 0.03 logMAR, respectively (mean ± SEM). Training did not affect distance acuities or Beery scores. Learning effects on uncrowded and crowded near visual acuities remained intact 6–12 months after training. We conclude that the pen-and-paper training specifically improves near visual acuities but does not transfer to distance acuities or fine motor skills. Improvements in near visual acuity are retained over time, bolstering its clinical value.

## Introduction

During the last decade, various visual training programs have been developed for children with low vision^1–4^. Although the precise nature of the intended effects differs, these training programmes share the common goal of improving vision (e.g., improving peripheral vision^3^ or central vision^1,2,4^). In traditional clinical practice, it is often believed that children with visual impairment generally are unresponsive to therapeutic techniques^3^. Perceptual learning refers to long-term improvements in perception of a stimulus after repeated exposure or experience with the stimulus. Training paradigms based on perceptual learning should not be confused with visual stimulation studies from the past^5^, because perceptual learning paradigms target behaviourally relevant sensory skills and are tailored to the performance level of the individual.

Evidence is accumulating that perceptual learning paradigms are an effective intervention for low vision in an increasing number of patient groups^1–4^. Individuals with low vision are more likely to benefit from perceptual learning, since training effects are typically larger for those with poorer initial performance^3,6–8^. Furthermore, it has been shown that there is broader transfer to untrained stimuli and tasks in these individuals^1,9,10^, making it more relevant for clinical rehabilitation interventions than one might expect from the task specificity of perceptual learning that is seen in individuals with normal vision.

**Figure 1:**
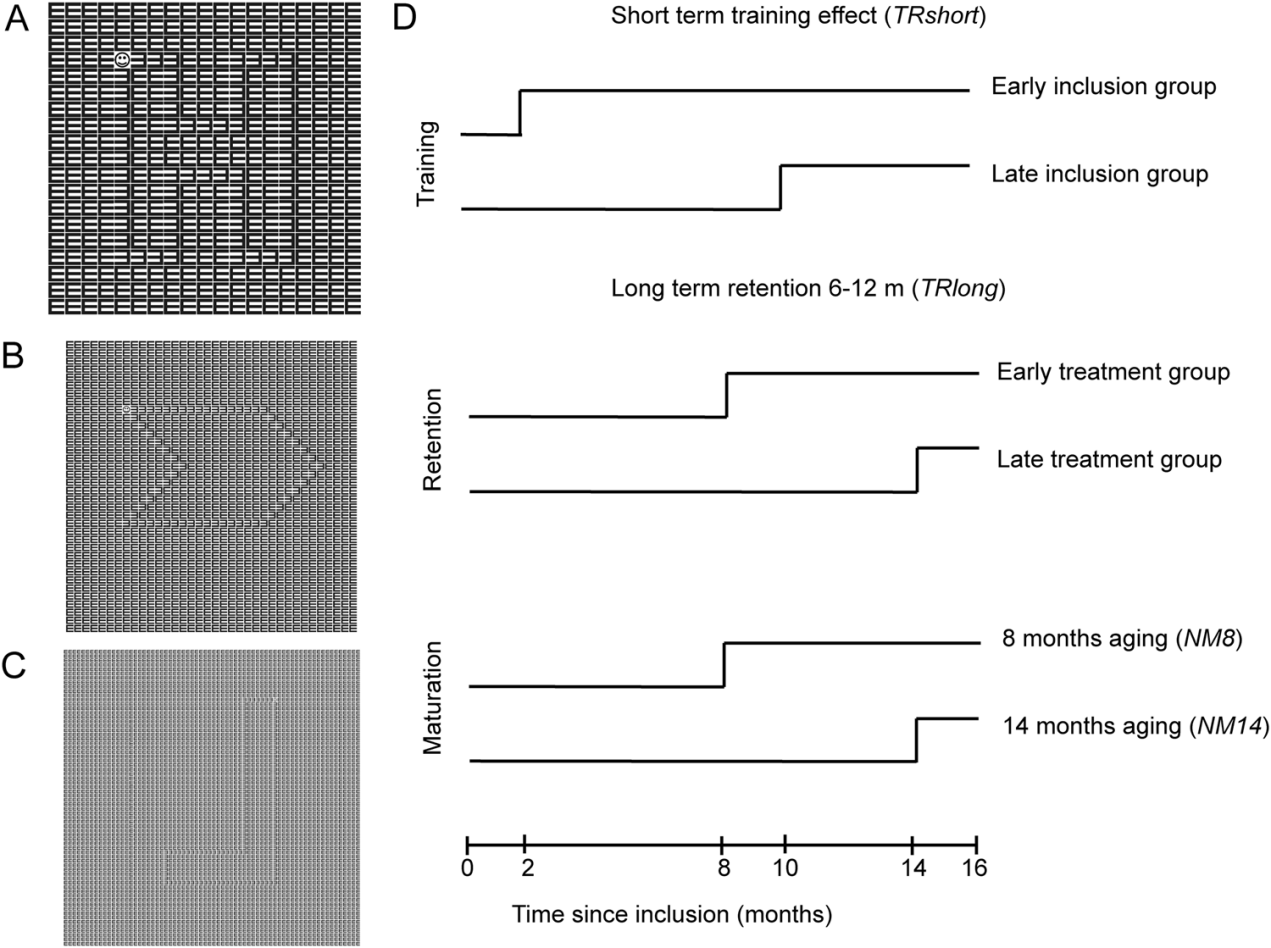
Example of the training material used in the crowded perceptual learning training with the large (**A**), intermediate (**B**) and small (**C**) optotypes sizes. (**D**) Visual representation of the step dummies in the design matrix of the ANCOVAs. Note that the time course of the predictors for the short- and long-term training effects in the early and late treatment group reflect the phased-treatment longitudinal design of our study. The two predictors for natural maturation necessarily follow the same time course in both groups.

We previously developed a training paradigm for young children with low vision that can improve visual acuity and reduce crowding^1^. Crowding refers to a decreased ability to identify objects in clutter and can be seen as a bottleneck in object recognition^2,11,12^. Its intensity can be expressed as the difference between crowded and uncrowded acuity (in logMAR). Crowding effects are often stronger in children^13^ (and adults^14^) with visual impairment. In addition, the age-related decrease observed in children with normal vision does not seem to occur in children with low vision. Our crowded perceptual learning training involves a pen-and-paper drawing game in which children are challenged to draw a figure by tracking a series of inversed Es embedded in an array of non-inversed Es to make the task more engaging for young children(Fig. 1A–C). The training still incorporates perceptual learning principles (improving visual performance by letting children work with near-threshold stimuli), but offers a more child-friendly approach to the conventional visual learning paradigms where subjects are seated at a fixed distance from the screen judging near identical stimuli over and over again. While the pen-and-paper training game results in short-term improvements on the trained task (i.e., better drawing performance and ability to discriminate smaller letters) and near visual acuity^1^ (NVA), it is still unclear whether 1) training effects transfer to distance visual acuity (DVA) and fine motor skills and 2) whether the training effects are long lasting.

The first goal of the present study was to test whether the near-vision learning effects of our crowded perceptual learning training transfer to visual acuity improvements at untrained, far viewing distances and whether the training improves fine visuomotor skills. The second goal was to measure the retention of its learning effects. There are persistent arguments that natural visual development improves visual acuity and reduces crowding in children as well – that is without training – and that this natural improvement obscures the benefit of visual training. Therefore, a phased-treatment longitudinal study design was used to disentangle long-term training effects from natural visual maturation in young children with low vision. We hypothesized that children show a training effect on top of the natural visual developmental effects. In addition, we expected that training effects would remain intact over a period of 6–12 months.

## Results

### Short and long term effects of training

The longitudinal data of our participants (n = 16, for participant characteristics see Table 1) collected at different time points before and after the training were analysed with repeated measures ANCOVAs. The ANCOVAs quantified the outcome changes with respect to the baseline (*Y*_0_) with four different step dummies (Fig. 1D) and included baseline performance (regression coefficient *β*_1_) as covariate. The step dummies were used to capture the initial short-term training effect (regression coefficient *β*_2_) and changes in this initial training effect in the long term (*β*_3_), as well as natural maturation occurring in the first eight months after inclusion (*β*_4_), and further maturation occurring after an additional six months (*β*_5_). Effects of procedural learning that might result from familiarity with the test procedure after the baseline measurements are captured by the intercept (*β*_0_).

**Table 1:** Characteristics of the participants in the early and late treatment group.

ID	Age at inclusion (years; months)	Clinical diagnosis	Baseline near crowded visual acuity (logMAR)	Binocular distance uncrowded visual acuity (logMAR)	Binocular distance crowded visual acuity (logMAR)	Nystagmus
**Early treatment group**						
1	5;7	Oculocutaneous albinism	0.50	0.18	0.52	Yes
2	7;6	Hypermetropia, astigmatism	0.50	0.40	0.51	No
3	5;9	Ocular albinism	0.90	0.66	0.92	No
4	6;6	High hypermetropia (>8D)	0.50	0.21	0.54	No
6	6;4	Opticopathy, both eyes	0.50	0.07	0.44	Yes
10	6;11	Oculocutaneous albinism	0.60	0.21	0.62	Yes
11	6;2	High myopia (>10D)	0.60	0.27	0.54	No
16	7;0	Opticopathy, both eyes	0.60	0.32	0.66	Yes
17	6;8	High hypermetropia, esotropia	0.60	0.04	0.49	Yes
**Late treatment group**						
5	6;4	Oculocutaneous albinism	1.20	1.06	1.25	Yes
7	8;0	Oculocutaneous albinism	0.60	0.51	0.82	Yes
9	6;6	Oculocutaneous albinism	0.50	0.51	0.89	Yes
12	6;5	Congenital nystagmus	0.50	0.38	0.52	Yes
13	6;3	Ocular albinism	0.60	0.34	0.74	Yes
14	5;10	Congenital nystagmus	0.90	0.49	0.92	Yes
18	7;7	Oculocutaneous albinism	0.60	0.15	0.46	Yes

#### Near visual acuity

**Figure 2:**
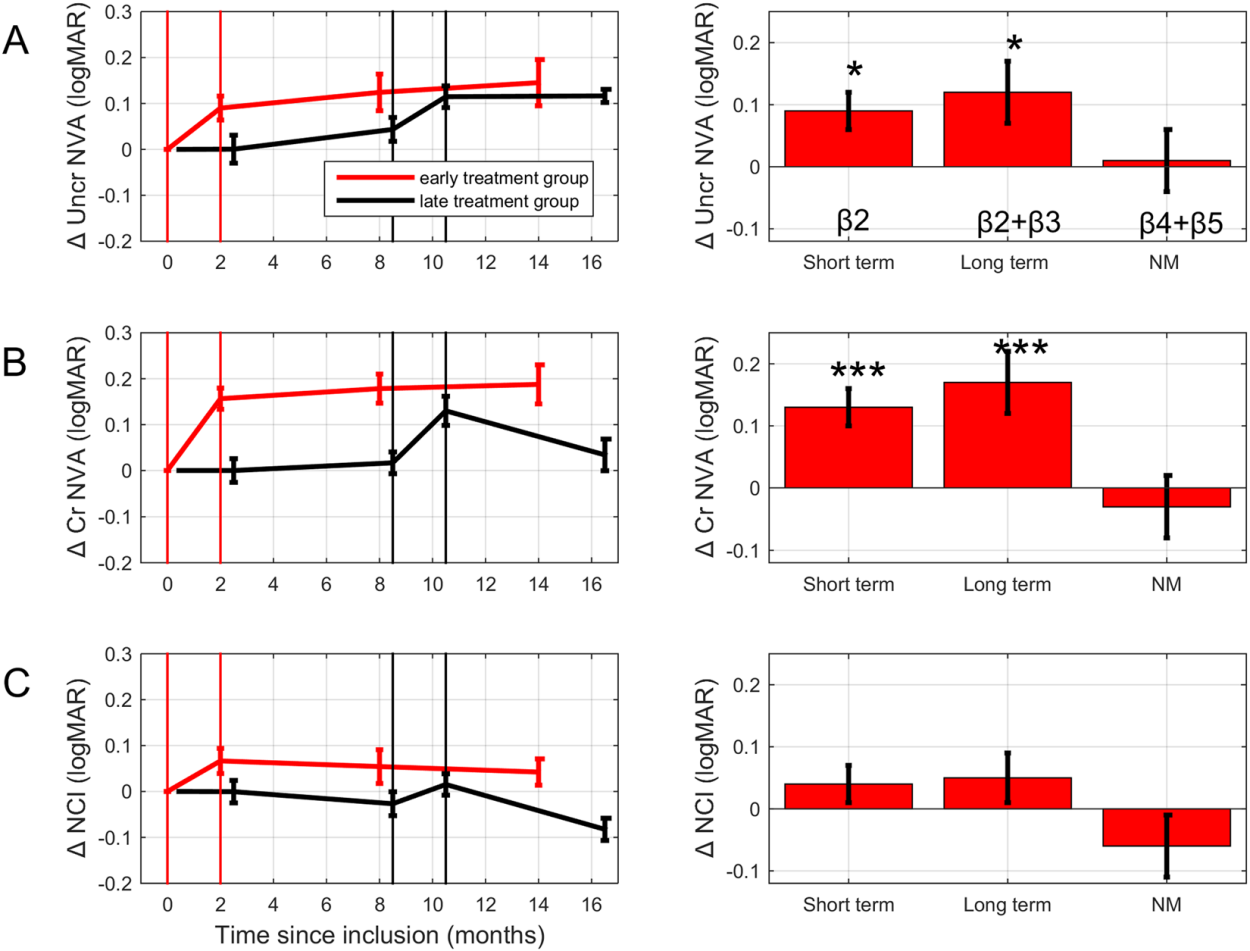
Effects of training on (**A**) uncrowded near visual acuity (NVA), (**B**) crowded NVA and (**C**) near crowding intensity (NCI). Left-hand panels: visual performance changes in the early (red) and late (black) treatment group as a function of time since inclusion. Positive values signify improvement. The data of the late treatment group has been shifted 0.5 months to the right for clarity. Right-hand panels: linear regression results quantifying the short-term training effects (*β*_2_), the long-term training effects (*β*_2_ + *β*_3_), and the effect of 12–14 months natural maturation (*β*_4_ + *β*_5_). Error bars: ± 1 SEM. *p < 0.05, ***p < 0.001.

The left-hand panels of Fig. 2 show the time course of the average changes in near visual acuity for the early (red) and late (black) intervention group. The right-hand panels show the average short-term and long-term training effects as well as the total effect of natural maturation as estimated from the children in both intervention groups via the ANCOVAs. Note, that training improved uncrowded NVA (*β*_2_ = 0.09 ± 0.03 logMAR, t(48) = 2.51, p = 0.016; Table 2; Fig. 2A) and that this initial training effect showed no significant decline in the following 6–12 months (*β*_3_ = 0.03 ± 0.04 logMAR, t(48) = 0.63, p > 0.5). If anything, the long-term training effect (*β*_2_ + *β*_3_) of 0.12 ± 0.05 logMAR tends to be larger than the short-term training effect (Fig. 2A, right). Because of our phased-intervention design, we can exclude that this due to regular natural maturation. Changes in uncrowded NVA due to natural maturation were very small (*β*_4_ and *β*_5_ < 0.02 logMAR). The total maturation effect after 14 months (*β*_4_ + *β*_5_) was only 0.01 ± 0.05 logMAR. Baseline acuity influenced the training effectivity: children with poorer baseline uncrowded NVA showed more improvement (*β*_1_ = 0.14 ± 0.06, t(48) = 2.42, p = 0.019).

**Table 2:** Parameters of the linear regression model describing NVA changes (logMAR; positive values indicate improvement).

Predictor	β (±SE)		t-Test	P-value	Partial correlation	R^2^	R^2^adj
**Uncrowded NVA changes**							
Intercept (logMAR)	−0.05 ± 0.04		−1.14	0.259	n.a.	0.26	0.18
Baseline (Y_0_)	0.14 ± 0.06		2.42	0.019	0.33		
TR_short_ (logMAR)	0.09 ± 0.03		2.51	0.016	0.34		
TR_long_ (logMAR)	0.03 ± 0.04		0.63	0.533	0.09		
NM_8_ (logMar)	0.01 ± 0.03		0.36	0.718	0.05		
NM_14_ (logMar)	0.00 ± 0.04		−0.06	0.950	−0.01		
**Crowded NVA changes**							
Intercept (logMAR)	−0.02 ± 0.04		−0.37	0.714	n.a.	0.38	0.31
Baseline (Y_0_)	0.07 ± 0.06		1.17	0.249	0.17		
TR_short_ (logMAR)	0.13 ± 0.03		3.86	<0.001	0.49		
TR_long_ (logMAR)	0.04 ± 0.04		0.90	0.371	0.13		
NM_8_ (logMar)	−0.01 ± 0.03		−0.32	0.747	−0.05		
NM_14_ (logMar)	−0.02 ± 0.04		−0.45	0.657	−0.06		
**Near crowding intensity changes**							
Intercept (logMAR)	−0.16 ± 0.04	−4.10		<0.001	n.a.	0.45	0.39
Baseline (Y_0_)	0.66 ± 0.13	5.27		<0.001	0.61		
TR_short_ (logMAR)	0.04 ± 0.03	1.32		0.194	0.19		
TR_long_ (logMAR)	0.01 ± 0.03	0.16		0.870	0.02		
NM_8_ (logMar)	−0.02 ± 0.03	−0.76		0.448	−0.11		
NM_14_ (logMar)	−0.04 ± 0.03	−1.45		0.153	−0.21		

The averaged data in Fig. 2B show a drop in crowded near visual acuity in the late intervention group 6 months after their training, suggesting that the retention of the gain in crowded near visual acuity might be different between the early and late intervention group. To test if this was the case, an interaction term was added to the regression model (Methods). It turned out, however, that this interaction term was not statistically significant (*β*_3*b*_ = 0.07 ± 0.05 logMAR, t(47) = 1.42, p = 0.163) and, therefore, it was left out of the model. Training was the only factor that explained variability in crowded NVA changes: children showed significant acuity improvements after training (*β*_2_ = 0.13 ± 0.03 logMAR, t(48) = 3.86, p < 0.001; Table 2, Fig. 2B) and these improvements showed no significant decline over the following 6–12 months (*β*_3_ = 0.04 ± 0.04 logMAR, t(48) = 0.90, p > 0.3). The average long-term training effect (*β*_2_ + *β*_3_) was 0.17 ± 0.05 logMAR indicating that the changes in crowded NVA were also retained. Natural maturation effects, on the other hand, were small and non-significant (*β*_4_ and *β*_5_ < 0.02 logMAR). The total maturation effect (*β*_4_ + *β*_5_) after 14 months was −0.03 ± 0.05 logMAR.

The crowding intensity at near viewing distance can be captured by subtracting the uncrowded from the crowded near visual acuity measure (in logMAR). Training and natural maturation did not have a significant impact on near crowding intensity (Table 2, Fig. 2C). The only factor that correlated with changes in near crowding intensity was baseline crowding intensity (*β*_1_ = 0.66 ± 0.13, t(48) = 5.27, p < 0.001).

#### Distance visual acuity

**Figure 3:**
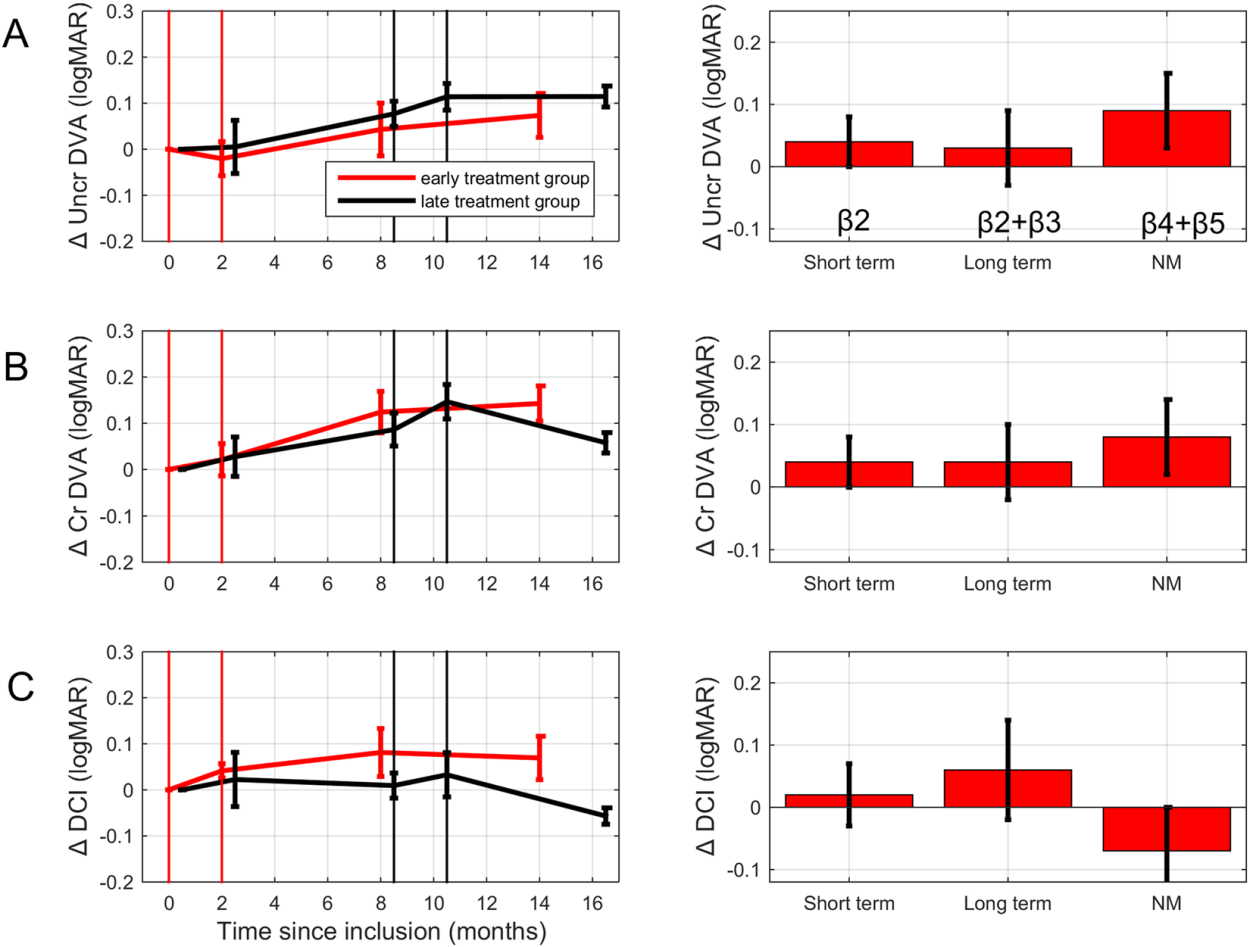
Effects of training on (**A**) uncrowded distance visual acuity (DVA), (**B**) crowded DVA, and (**C**) distance crowding intensity (DCI). The red lines display data collected in the early treatment group and black lines represent data collected in the late treatment group. The data of the late treatment group has been shifted 0.5 months to the right for clarity. Right-hand panels: linear regression results for the short-term training effects (*β*_2_), the long-term training effects (*β*_2_ + *β*_3_), and the effect of 12–14 months natural maturation (*β*_4_ + *β*_5_). Error bars: ±1 SEM. No significant changes were found.

Figure 3 shows the results for distance visual acuity (same format as Fig. 2). In contrast to the effects of training on NVA, there was no significant influence of training on uncrowded DVA (*β*_2_ = 0.04 ± 0.04 logMAR, t(48) = 1.08, p > 0.2; see Table 3, Fig. 3A). Natural maturation tended to improve uncrowded DVA in the first 8 months (*β*_4_ = 0.08 ± 0.04 logMAR, t(48) = 1.84, p = 0.072), but total maturation effects after 14 months (*β*_4_ + *β*_5_ = 0.09 ± 0.06 logMAR) were not significant.

**Table 3:** Parameters of the linear regression model describing DVA changes (logMAR; positive values indicate improvement).

Predictor	β (±SE)	t-Test	P-value	Partial correlation	R^2^	R^2^adj
**Uncrowded DVA changes**						
Intercept (logMAR)	−0.14 ± 0.04	−3.17	0.003	n.a.	0.39	0.32
Baseline (Y_0_)	0.29 ± 0.06	4.50	<0.001	0.54		
TR_short_ (logMAR)	0.04 ± 0.04	1.08	0.287	0.15		
TR_long_ (logMAR)	−0.01 ± 0.05	−0.18	0.860	−0.03		
NM_8_ (logMar)	0.08 ± 0.04	1.84	0.072	0.26		
NM_14_ (logMar)	0.01 ± 0.05	0.31	0.761	0.04		
**Crowded DVA changes**						
Intercept (logMAR)	−0.02 ± 0.07	−0.32	0.751	n.a.	0.16	0.07
Baseline (Y_0_)	0.03 ± 0.07	0.35	0.727	0.05		
TR_short_ (logMAR)	0.04 ± 0.04	0.85	0.402	0.12		
TR_long_ (logMAR)	0.00 ± 0.05	−0.09	0.930	−0.01		
NM_8_ (logMar)	0.10 ± 0.04	2.30	0.026	0.31		
NM_14_ (logMar)	−0.02 ± 0.05	−0.37	0.712	−0.05		
**Distance crowding intensity changes**						
Intercept (logMAR)	−0.07 ± 0.07	−1.03	0.310	n.a.	0.08	−0.01
Baseline (Y_0_)	0.28 ± 0.18	1.59	0.118	0.22		
TR_short_ (logMAR)	0.02 ± 0.05	0.47	0.639	0.07		
TR_long_ (logMAR)	0.04 ± 0.06	0.77	0.443	0.11		
NM_8_ (logMar)	−0.01 ± 0.05	−0.14	0.887	−0.02		
NM_14_ (logMar)	−0.06 ± 0.05	−1.13	0.262	−0.16		

Variability in crowded DVA changes could not be explained by training effects either (*β*_2_ = 0.04 ± 0.04, Table 3; Fig. 3B). Natural maturation did have a significant impact on crowded DVA: it was improved 8 months after inclusion (*β*_4_ = 0.10 ± 0.04 logMAR t(48) = 2.30, p = 0.026). Fourteen months after inclusion, however, the total maturation effect was no longer significant (*β*_4_ + *β*_5_ = 0.08 ± 0.06 logMAR). Distance crowding intensity (i.e., crowded DVA minus uncrowded DVA in logMAR) was not significantly influenced by any of the experimental factors or maturation (F(6,48) = 0.88, p > 0.5, Table 3; Fig. 3C, *β*_4_ + *β*_5_ = −0.07 ± 0.07 logMAR).

#### Fine motor skills

**Figure 4:**
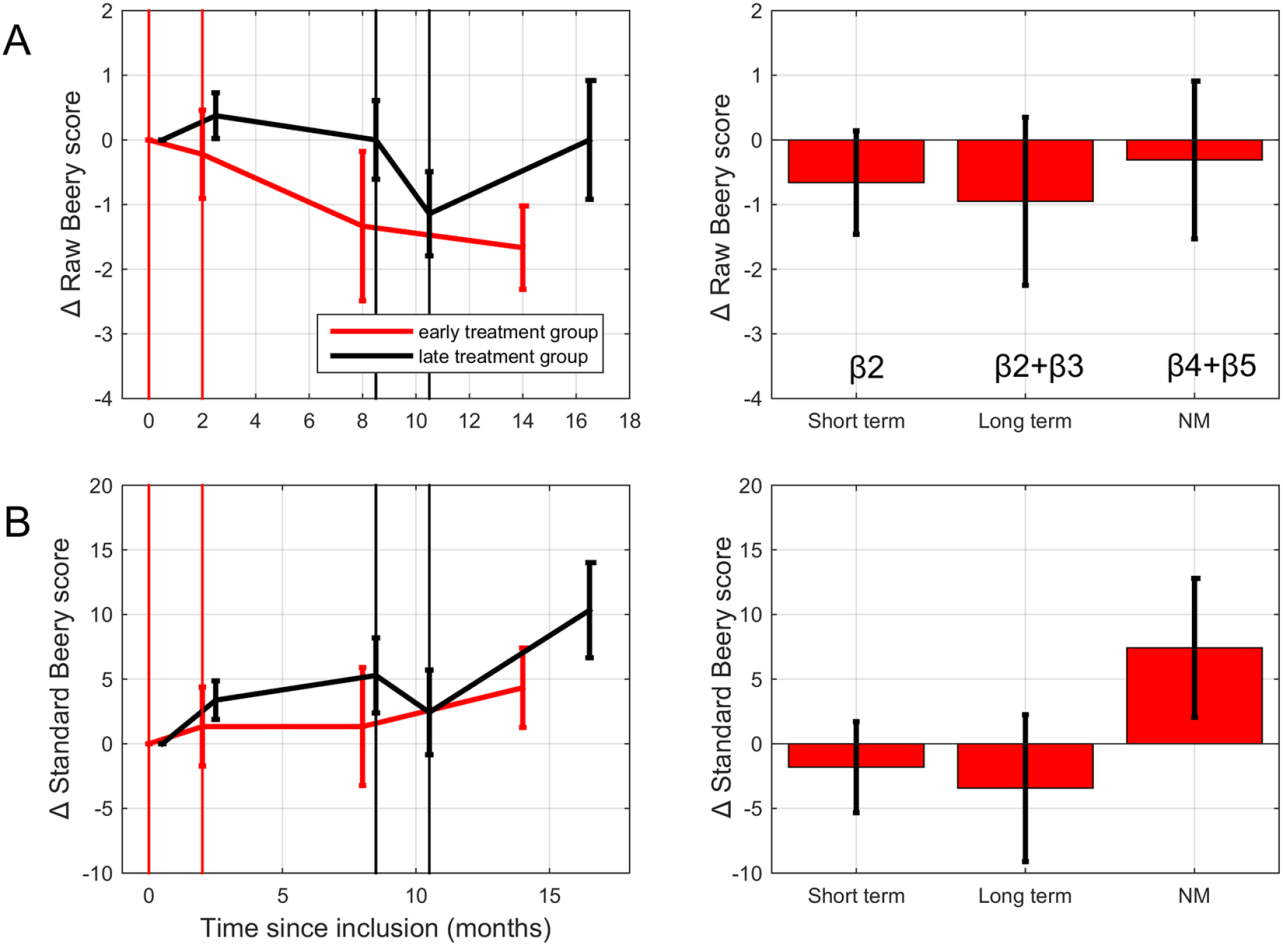
Effects of training on raw (**A**) and standard (**B**) Beery Motor Control scores. Red: early treatment group. Black: late treatment group. Data of the late treatment group has been shifted 0.5 months to the right for illustration purposes. Negative values signify improvement. Right-hand panels: linear regression results for the short-term training effects (*β*_2_), the long-term training effects (*β*_2_ + *β*_3_), and the effect of 12–14 months natural maturation (*β*_4_ + *β*_5_). Error bars: ±1 SEM. No significant changes were found.

Raw and standard Beery Scores were not influenced by the experimental factors (see Table 4, Fig. 4A,B). The regression models did not account for variability in these outcome measures (F(6,48) = 1.82, p = 0.128, and F(6,48) = 1.20, p = 0.324, respectively).

**Table 4:** Parameters of the linear regression model describing Beery score changes (negative values indicate improvement).

Predictor	β (±SE)	t-Test	P-value	Partial correlation	R^2^	R^2^adj
**Raw Beery score changes**						
Intercept	−3.53 ± 1.76	−2.01	0.050	n.a.	0.16	0.07
Baseline (Y_0_)	0.24 ± 0.10	2.41	0.020	0.33		
TR_short_	−0.66 ± 0.80	−0.82	0.417	−0.12		
TR_long_	−0.29 ± 1.02	−0.29	0.775	−0.04		
NM_8_	−0.63 ± 0.80	−0.79	0.435	−0.11		
NM_14_	0.32 ± 0.92	0.35	0.728	0.05		
**Standard Beery score changes**						
Intercept	−18.45 ± 12.63	−1.46	0.150	n.a.	0.11	0.02
Baseline (Y_0_)	0.24 ± 0.14	1.74	0.089	0.24		
TR_short_	−1.81 ± 3.52	−0.52	0.608	−0.07		
TR_long_	−1.55 ± 4.46	−0.35	0.729	0.05		
NM_8_	1.96 ± 3.52	0.56	0.581	0.08		
NM_14_	5.46 ± 4.05	1.35	0.184	0.19

### Test-retest variability

Test-retest differences and test-retest variability was evaluated by determining test-retest differences (mean ± SEM) and the limit of agreement (LOA, standard deviation difference*1.96) between the first and second measure collected in the late treatment group, i.e., 8 and 6 months before they started the training. The interval between the first and second measure was 6–8 weeks. The test-retest differences for uncrowded NVA was 0.01 ± 0.03 logMAR (LOA ± 0.17 logMAR). For the crowded NVA measure the test-retest difference was 0.02 ± 0.02 logMAR (LOA ± 0.14 logMAR). These measures thus show good agreement between these repeated NVA measures. For the DVA measures collected with the FrACT (24 trials per run with 6 ‘easy optotypes’, 4AFC), test-retest variability was larger. The test-retest difference for uncrowded DVA was −0.01 ± 0.07 logMAR (LOA ± 0.36 logMAR). For the crowded DVA, it was 0.01 ± 0.05 logMAR (LOA ± 0.25 logMAR). Test-retest differences for the Beery raw and standard scores were 0.14 ± 0.32 (total number of figures drawn without making errors) and 2.29 ± 1.24 (where standard scores have a mean of 100 and a standard deviation of 15) (LOA ± 1.76 and ± 6.86, resp.).

## Discussion

The results of the present study indicate that six weeks of near vision training with a crowded letter configuration resulted in near visual acuity improvements. In addition, our present findings indicate that the training effects on near vision are fully retained over a period of 6–12 months. The observed improvements were very much in line with the short-term effects reported in our earlier study (~0.10–0.15 logMAR)^1^. We found no transfer of learning effects to distance visual acuity improvements or fine motor skills improvements. Below we elaborate on these outcomes.

### Transfer of training effects

At first glance, training effects appear to be distance-dependent. It should be noticed, however, that test-retest variability of the distance visual acuity measures (LOA ± 0.25 and ± 0.36 logMAR) was considerably larger than variability of near visual acuity measures (LOA ± 0.17 and ± 0.14 logMAR). This larger test-retest variability makes it harder to detect significant distance visual acuity changes in a small group of participants. In addition to the larger test-retest variability of the distance visual acuity measures, training-induced improvements in crowded acuity were smaller for distance than near visual acuity (e.g., 0.13 ± 0.03 logMAR crowded NVA versus 0.04 ± 0.04 logMAR for crowded DVA).

Another explanation for the absence of a significant training effect on DVA is that NVA acuity improvements might be the result of improvements in the control of accommodation and/or vergence. Near visual acuity can be seen as the joint product of distance visual acuity, refraction correction, accommodative power, and vergence accuracy whereas distance visual acuity is thought of as an ocular property independent of accommodative power and accuracy^15,16^. Participants all wore their glasses during training and testing, and did not change refraction correction over the course of the study. Thus, refraction correction cannot explain the differences between near and distance visual acuity changes. Vergence and accommodation systems are cross-linked; stimuli to either system (disparity or blur) activate both systems^17^. The training put a high demand on accommodation accuracy and the accuracy of vergence eye movements as the children had to discriminate near-threshold letters at a short viewing distance. In adults with presbyopia, near vision training does not affect accommodative power and accuracy^18^. The accommodative power in the subjects tested in the presbyopia study was about 0.5 dioptres. In school-aged children, however, it is around 15 dioptres^16^. It is likely that the accommodative system is more plastic and susceptible to training effects at a younger age.

The majority of the children that were included could not read yet (11/16). Nevertheless, because training effects seem to be distance-dependent, we were curious whether training might have influenced the reading performance of the ones that could read at the time of their inclusion. The mean (±SEM) reading acuity tended to improve by 0.06 ± 0.08 logMAR (paired t-test, p > 0.20) and maximum reading speed improved with a median of 9 words per minute after training (Wilcoxon signed rank p = 0.125), but with only 5 young children in this group, for whom reading performance is also highly variable, the statistical power was too low to allow for reliable conclusions.

Finally, there is evidence that a combination of training aspects (a broad range of frequencies and stimulus orientations) and more extensive training results in broader transfer^19^ than training paradigms using only one spatial frequency and or orientation^20,21^. Learning transfer might be boosted by including more stimulus orientations and larger viewing distances.

### Maturation effects

The average age at the time of inclusion was 6 years and 7 months. A previous cross-sectional study in typically developing children reported single letter acuity improvements with age from an average of 0.02 logMAR at 5 years to −0.09 logMAR at 8 years^22^, i.e., a 0.11 logMAR acuity change in 3 years. Thus, it is not surprising that the average acuity changes that resulted from natural maturation over the course of our 14–16 month study period were all smaller than 0.1 logMAR.

### Retention of training effects

A previous perceptual learning study in adults with amblyopia reported retention of improvements in crowded NVA of 91 ± 16% over a period of 3–14 months after training^23^. Our phased-treatment longitudinal design allowed us to dissociate the long-term retention of the learning effects from natural maturation that could occur naturally over such a prolonged period of time in young children. Short-term training effects were 0.09 ± 0.03 logMAR for uncrowded near visual acuity and 0.13 ± 0.03 logMAR for crowded near visual acuity. The long-term training effects corrected for natural maturation were 0.12 ± 0.05 logMAR and 0.17 ± 0.05 logMAR over a period of 6 months after training (i.e., *β*_2_ + *β*_3_) for uncrowded and crowded near visual acuity, respectively.

The lack of significant natural maturation effects on near visual acuity (between 0.01 and 0.04 logMAR) further bolters our conclusion that developmental changes in NVA cannot account for the observed long-term improvements. Our results suggest that retention was much better than the 91% described for adults with amblyopia. This follows from our finding that the betas of the term that captures the after-effect of the training on the NVA measures (i.e., the *β*_3_*s*) were always positive. Although not significantly > 0 statistically, this trend hints at the possibility that our training might facilitate natural maturation after the intervention.

## Conclusions

Our longitudinal study provides new insights into the impact of a near vision training and time on visual acuity and fine motor skills in 4- to 8-year-old visually impaired children. It shows that the near vision pen-and-paper training yields long lasting near visual acuity improvements on top of natural maturation. Distance visual acuity and Beery motor control scores remain unaltered. More research is needed to evaluate possible transfer to reading performance and to test the idea that visual perceptual learning in young children with low vision might facilitate their natural visual development on top of the direct training-induced NVA improvements.

## Methods

### Subjects

Sixteen children participated in the study (8 boys, 8 girls). Inclusion criteria were age between 4 and 8 years, a crowded near visual acuity equal to or better than 1.3 logMAR (20/400 or 0.05 decimal) and weaker than or equal to 0.3 logMAR (20/40 or 0.50 decimal). In addition, children had to have a normal birth-weight, born at term without perinatal complications, show normal development and no additional impairments on top of their visual impairment. Informed consent was obtained from the parents of all participating children after they were given explanation of the nature and possible consequences of the study. The local ethics committee (CMO Arnhem-Nijmegen, The Netherlands, protocol ID NL59403.091.16) approved the study protocol and the study was conducted in accordance with the principles of the Declaration of Helsinki.

After inclusion, children were randomly assigned to the early and late treatment group using a permuted-blocks randomization schedule, stratified by age. Mean age and baseline near visual acuity of participants in the early and late treatment group did not differ significantly. Mean age was 77.9 ± 7.3 months in the early treatment group and 80.4 ± 9.4 months in the late treatment group. Mean crowded NVA was 0.58 ± 0.14 logMAR in the early and 0.72 ± 0.25 logMAR in the late treatment group. Because children were included based on their crowded NVA, groups comprised children with different diagnoses (Table 1).

### Training

The training paradigm was inspired by the Eriksen flanker task^24^. Children were instructed to draw a line through a trail of inversed Es embedded in a 145 × 145 mm grid filled with non-inversed Es (high target-distractor similarity evokes crowding)^25^. They had to start and end at the smiling face (Fig. 1A). Edge-to-edge optotype spacing was kept fixed at 0.3 mm (0.04° at 40 cm). By drawing a line through the trail of the inversed E’s, children ended up with a figure. All children started their training with optotypes of 7.0 mm, which equals 4 M. One Sloan M-unit is the optotype size that corresponds to a visual angle of 5 minute of arc at 1 meter so the same visual angle applies for 2 M-units at 2 meters, 3 M-units at 3 meters, etc.^26^. During training, children could adopt a self-chosen viewing distance. If children were able to draw a figure without errors and could complete 12 trials in a 30-minute training session, they progressed to booklets with optotypes of 3.5 mm (2 M), and eventually 1.75 mm (1 M) on subsequent training sessions (Fig. 1B,C). Children performed the training under supervision of an occupational therapist.

### Procedure

Binocular uncrowded and crowded distance visual acuity were measured at 3 meters with the Freiburg Visual Acuity Test^27^ (FrACT, with a single optotype presentation and an inter optotype spacing of 2.6 arc minutes for the crowded measurement using 24 trials per run with 6 ‘easy optotypes’). Near visual acuity was measured at 0.4 meters with the LEA-version of the C-test^13^. Crowding intensities were determined from the difference between crowded and uncrowded acuity measured (in logMAR) at the same distance. Fine motor skills were evaluated with the subtest Motor Control of the Beery VMI, because this test assesses drawing accuracy. Reading measures, obtained with the Radner test^28^, are not presented because of the small number of children that could read at the time of their inclusion (n = 5).

In the early treatment group, outcome measures were collected at 0 months (before training), at 2 months (directly after training), at 8 months (6 months after training) and at 14 months (12 months after training) since inclusion. In the late treatment group, training started 8 months after inclusion and three baseline measurements were performed before the training to provide developmental and test-retest control data. Additional measurements were taken at 0 and 6 months post-training to evaluate short- and long-term training effects in this group. This phased treatment scheme was adopted in order to collect control data for the first three measurements in the early treatment group.

Training started within two weeks after the (last) pretest. Children trained twice per week for six consecutive weeks resulting in 12 training sessions (30 minutes per training session).

### Statistical analysis

We analysed the observed changes in visual acuity and fine motor skills with respect to their baseline values using repeated measures ANCOVA (MATLAB version 2014b, MathWorks, Inc., Natick, MD). The regression always included five predictors (Fig. 1D). The first predictor, baseline performance (*Y*_0_, e.g., VA in logMAR collected at 0 months since inclusion), was included as covariate. The second was a step dummy to quantify short-term performance changes due to training (*TR*_*short*_, 0 for all pre-training measurements, and 1 from the first post-training measurement onwards, i.e., from t = 2 and t = 10 months for the early and late treatment group, respectively). The third was a step dummy to quantify any changes in the initial training effect in the long term (*TR*_*long*_, 1 from t = 8 or t = 16 months follow-up in the early and late treatment group, respectively, and 0 prior to that). The remaining two predictors were step dummies to describe possible changes due to natural maturation (*NM*_8_ and *NM*_14_, steps from 0 to 1 at t = 8 and t = 14 months, respectively, for both treatment groups). This simple, stepwise maturation model assumes that natural changes over a period of 2 month are negligible and that changes over a 12-month period need not be linear. This resulted in the following regression equation for performance changes, Δ*Y*, as a function of time, *t*:

$$\varDelta Y(t)={\beta }_{0}+{\beta }_{1}\cdot {Y}_{0}+{\beta }_{2}\cdot T{R}_{short}(t;G)+{\beta }_{3}\cdot T{R}_{long}(t;G)+{\beta }_{4}\cdot N{M}_{8}(t)+{\beta }_{5}\cdot N{M}_{14}(t)$$

where *G* denotes the treatment group (1 = early, 0 = late) and positive Δ*Y* values reflect improvements with respect to baseline. In this model, the effect of baseline acuity (*β*_1_) and the natural maturation effects (*β*_4_ and *β*_5_) are within subjects factors. The short term training effect (*β*_2_) and the change in this initial training effect after 6–12 months (*β*_3_) are mixed factors, since the timing of the intervention is different for the two treatment groups. The net long-term training effects are given by the sum of *β*_2_ and *β*_3_. Thus, if short-term training effects are retained over time, *β*_3_ (*TR*_*long*_) will be zero (full retention) or perhaps even positive if the training treatment facilitates the subsequent natural maturation. Conversely, if the training effects subside over time, *β*_3_ will be negative. More specifically, a value of *β*_3_ = −*β*_2_ would indicate that there is no long-term retention whatsoever. *β*_4_ represents the maturation occurring over 8 months while the sum *β*_4_ + *β*_5_ relfects the total maturation occurring over 12–14 months. The model intercept, *β*_0_, reflects non-specific changes in the outcomes that could result, e.g., from increased familiarity with the test procedure after the baseline measurements.

The regression model described above assumes that short- and long-term training effects are similar in the early and late treatment group. In case the averaged group data suggested a difference between long-term changes after the early and late treatment, this potential difference between the two groups was modelled with an additional interaction term in the following way:

$$\begin{array}{ccc}\varDelta Y(t) & = & {\beta }_{0}+{\beta }_{1}\cdot {Y}_{0}+{\beta }_{2}\cdot T{R}_{short}(t;G)+{\beta }_{3a}\cdot T{R}_{long}(t;G)\\  &  & +\,{\beta }_{3b}\cdot T{R}_{long}\cdot G+{\beta }_{4}\cdot N{M}_{8}(t)+{\beta }_{5}\cdot N{M}_{14}(t)\end{array}$$

For the test-retest analysis, we computed the mean difference between repeated measurements at t = 0 and t = 2 months in the late treatment group, i.e., before they received training. Test-retest reliability was expressed as the 95% limit of agreement between the measures at these time points (LOA, i.e. standard deviation difference*1.96). For the FrACT, the mean difference ± LOA for adults with (corrected to) normal vision has been reported to be 0.01 ± 0.10 logMAR (using 24 presentations and 8 possible C-orientations^29^) and 0.03 ± 0.20 logMAR (18 presentations, 8 possible C-orientations^30^). This has been interpreted as indicative for ‘high agreement’ and ‘room for improvement’, respectively. Unless specified otherwise, values are reported as mean ± standard error of the mean (SEM).

## Data Availability

Requests for materials and data should be addressed to B.H. (b.huurneman@donders.ru.nl).
